# Geometric Features of the Pial Arteriolar Networks in Spontaneous Hypertensive Rats: A Crucial Aspect Underlying the Blood Flow Regulation

**DOI:** 10.3389/fphys.2021.664683

**Published:** 2021-07-05

**Authors:** Dominga Lapi, Martina Di Maro, Nicola Serao, Martina Chiurazzi, Maurizio Varanini, Lina Sabatino, Rossana Scuri, Antonio Colantuoni, Bruna Guida

**Affiliations:** ^1^Department of Biology, University of Pisa, Pisa, Italy; ^2^Department of Clinical Medicine and Surgery, University of Naples “Federico II”, Naples, Italy; ^3^Institute of Clinical Physiology, National Council of Research (CNR), Pisa, Italy; ^4^Department of Sciences and Technologies, Sannio University, Benevento, Italy; ^5^Department of Translational Research and New Technologies in Medicine and Surgery, University of Pisa, Pisa, Italy

**Keywords:** hypertension, pial microcirculation, strahler scheme, rhythmic diameter changes, spontaneous hypertensive rat (SHR)

## Abstract

**Background:**

Several studies indicate that hypertension causes major changes in the structure of the vessel wall by affecting the regulation of blood supply to the tissues. Recently, it has been observed that capillary blood flow is also considerably influenced by the structural arrangement of the microvascular networks that undergo rarefaction (reduction of the perfused vessel number). Therefore, this study aimed to assess the geometric arrangements of the pial arteriolar networks and the arteriolar rhythmic diameter changes in spontaneously hypertensive rats (SHRs).

**Methods:**

Fluorescence microscopy was utilized to observe *in vivo* the pial microcirculation through a closed cranial window. Pial arterioles were classified according to Strahler’s method. The arteriolar rhythmic diameter changes were evaluated by a generalization short-time Fourier transform.

**Result:**

Young SHRs showed four orders of vessels while the adult ones only three orders. The diameter, length, and branching number obeyed Horton’s law; therefore, the vessels were distributed in a fractal manner. Larger arterioles showed more asymmetrical branches than did the smaller ones in young SHRs, while in adult SHRs smaller vessels presented asymmetrical branchings. In adult SHRs, there was a significant reduction in the cross-sectional area compared with the young SHRs: this implies an increase in peripheral resistance. Young and adult age-matched normotensive rats did not show significant alterations in the geometric arteriolar arrangement with advancing age, both had four orders of arteriolar vessels, and the peripheral resistance did not change significantly. Conversely, the frequency components evaluated in arteriolar rhythmic diameter changes of young and adult SHRs showed significant differences because of a reduction in the frequency components related to endothelial activity detected in adult SHRs.

**Conclusion:**

In conclusion, hypertension progressively causes changes in the microarchitecture of the arteriolar networks with a smaller number of vessels and consequent reduced conductivity, characteristic of rarefaction. This was accompanied by a reduction in the formation and release of independent and dependent – endothelial nitric oxide components regulating arterial vasomotion.

## Introduction

Cerebral microcirculation has unique features because it is vulnerable under physiopathological conditions, such as hypertension and aging (Hutchins et al., 1996; Levy et al., 2001; Bohlen, 2009). A great number of studies, indeed, has shown that hypertension is a major risk factor for stroke; moreover, an increase in systemic arterial blood pressure has been implicated in vascular cognitive impairment and Alzheimer’s disease: both conditions have a significant vascular pathology (Schrader, 2009; Veglio et al., 2009; Iadecola and Gottesman, 2019; Wajngarten and Sampaio, 2019). In hypertension, the structure and function of microcirculation undergo substantial alterations: the mechanisms of tone regulation (myogenic response) may be impaired, leading to increased vasoconstriction or reduced vasodilating responses (Vicaut, 1999; Feihl et al., 2006). Furthermore, there is thickening of the arteriolar wall or changes in the architecture of the arteriolar networks are involved (Koller, 2002).

It has been suggested that microvascular networks are depleted of vessels (rarefaction) with consequent reduction of the blood supply to the tissues (Boegehold et al., 1991). This feature is critical because the transport of oxygen and nutrients to the cells is over a physical distance; hence, the system of vessels must not only distribute blood effectively but also efficiently, so that the work of transport is not too expensive in terms of energy or time (Prewitt et al., 1982; Sokolova et al., 1985; Baumbach and Heistad, 1989). This mechanism requires a real compromise between regulation of the arteriolar tone and geometric arrangement of the microvascular system.

Many clinical and experimental data clearly show that hypertension causes modifications of the vascular system involving the arterioles that are responsible for the fine regulation of capillary perfusion (Renna et al., 2013; Laurent and Boutouyrie, 2015; Martinez-Quinones et al., 2018).

Alterations occurring in primary and secondary arterial hypertension have been identified, and all the reported data support that the major hemodynamic alterations consist in increased vascular resistance of smaller arterioles, while microvascular rarefaction has been observed in the very early stages of hypertension development (Rizzoni et al., 1994; Antonios et al., 1999).

Therefore, the aim of the present study was to assess the geometric arrangements of the pial arteriolar networks and the arteriolar rhythmic diameter changes in spontaneously hypertensive rats (SHRs). The investigation was carried out in young and adult SHRs because these animal models have interesting features, namely, that of developing high blood pressure levels progressively with advancing age. The principal purpose was to enumerate the number of vessels, but also the types of vessels, their length, and branchings, in order to geometrically characterize all arteriolar networks. Moreover, we recorded the arteriolar rhythmic diameter changes to evaluate the frequency components modulating arteriolar vasomotion during the development of hypertension. We tried to clarify all these factors that are known to limit the autoregulation of cerebral blood flow and increase susceptibility to pathological events.

## Materials and Methods

The animals used were treated according to the rules dictated by the Guide for the Care and Use of Laboratory Animals of the National Institutes of Health. The protocol was approved by the Committee on the Ethics of Animal Experiments of the University of Pisa and the Italian Health Ministry (permit number: 156/2017-PR).

The experiments were carried out on young (*n* = 15, systolic blood pressure = 108 ± 2 mmHg, diastolic blood pressure = 85 ± 3 mmHg, age = 3–4 months) and adult (*n* = 15, systolic blood pressure = 220 ± 8 mmHg, diastolic blood pressure = 170 ± 5 mmHg, age = 8–10 months) spontaneously hypertensive male rats.

These two experimental groups were compared with two age-matched groups consisting of young (*n* = 15, systolic blood pressure = 110 ± 3 mmHg, diastolic blood pressure = 80 ± 4 mmHg, age = 3–4 months) and adult (*n* = 15, systolic blood pressure = 115 ± 5 mmHg, diastolic blood pressure = 90 ± 3 mmHg, age = 8–10 months) normotensive male Wistar rats.

All animals were kept at constant temperature (24 ± 1°C) and humidity (60 ± 5%), with free access to food and water, and subjected to a 12-h artificial light–dark circadian cycle (12 h light/12 h darkness).

### Physiological Characteristics of SHRs

SHRs, the most widely used hypertensive rat model, were obtained by selecting hypertensive traits with Mendelian transmission during the crossing of Wistar rats. SHRs were first obtained in 1963 by Okamoto and Aoki (1963) by mating Wistar–Kyoto rats with high levels of arterial blood pressure. As in humans, SHRs develop progressive hypertension, characterized by an increase in cardiac output and peripheral resistance. When the cardiac output stabilizes (at 5–6 weeks of life), the vessels are hypertrophic, causing a further increase in peripheral resistance (Bianchi et al., 1974; Smith and Hutchins, 1979), with the arterial blood pressure continuously increasing until it reaches a plateau (after 40–60 days). Moreover, SHRs develop glomerulosclerosis, proteinuria, interstitial fibrosis, as well as cardiovascular diseases, such as cardiac hypertrophy (Ofstad and Iversen, 2005). Between 14 and 15 weeks, renal disease also develops, with a decrease in glomerular filtration by 20–30% of the baseline (Reckelhoff et al., 1997).

### Surgical Procedure

Animals were anesthetized by intraperitoneal injection of α-chloralose at a dosage of 50 mg/kg of body weight in 0.4 ml of physiological solution (0.9% sodium chloride: 1,000 ml contain 9 g of sodium chloride, 154 mEq/L water each of Na^+^ and Cl^–^ for injections, pH 4.5–7.0, osmolarity = ∼308 mOsm/l). They were maintained under anesthesia for the duration of the experiments by i.v. administration of α-chloralose at a dosage of 20 mg/kg of body weight (0.2 ml of physiological solution), approximately every 50 min.

Successively, anesthetized rats were tracheotomized and intubated. A catheter was introduced into the femoral vein for injection of the fluorescent tracer and the maintenance anesthetic. A second catheter was inserted into the femoral artery to measure arterial blood pressure. During the experiment, the animal’s body temperature was maintained at 37.0 ± 0.5°C by keeping the rat on a specially heated stereotaxic support (Lapi et al., 2012).

### Blood Pressure Measurements

Arterial blood pressure was measured when the rats were awake. Systolic and diastolic blood pressures were acquired by means of the non-invasive Mouse and Rat Tail Cuff Method Blood Pressure Systems (MRBP; IITC, Life Science Inc., Los Angeles, CA, United States), which allowed us to measure the arterial blood pressure from the animal’s tail. The animals were introduced in a Plexiglas tube, maintained in a prone position with the tail inserted in a cuff equipped with a transducer.

### Evaluation of Microvascular Parameters by Fluorescence Microscopy Technique

To observe pial microcirculation, a cranial window was inserted at the parietal bone level (2 mm posterior to the bregma and 3 mm from the midline) (Mayhan and Heistad, 1985) in each rat. An incision of about 1 cm was made on the skin, thus exposing the skull. The skin flaps were retracted with sutures to form a well for the perfusion fluid. Craniotomy was then performed and the dura mater was incised to expose the vessels. Two conduits, one inlet and one discharge, maintained the constant flow and outflow of artificial cerebrospinal fluid, bathing the brain surface homogeneously, for the entire duration of the experiment. We evaluated the number and order of vessels in a window of 500 × 500 μm in all groups of animals.

The diameters and lengths of the pial vessels were measured using an appropriate software (MIP-CNR, Pisa, Italy) that allows us to visualize portions of the cranial window. Firstly, terminal arterioles were identified, which give rise to capillaries; subsequently, higher-order arterioles were identified. For each vessel considered, the diameter was expressed as the average ± standard error of the mean (SEM) of three sizes of caliber carried out in adjacent areas of the vessel itself; the length was measured with repeated and successive measurements on the course of the vessel between one branch and the next. By assembling the photographs of the vessels taken directly from the monitor in stop-frame conditions and reporting the lengths and diameters thus obtained on them, it was possible to construct the map of each microvascular network studied. The order was assigned to a vessel according to Strahler’s scheme (Jiang et al., 1994), modified according to the diameter, in accordance with the method previously proposed (Kassab et al., 1993; Jiang et al., 1994; Kassab et al., 1994; Lapi et al., 2008). Assuming that D*n* and Δ*n* are the mean and the standard deviation of any arbitrary order *n*, respectively, it is possible to define a range of values around D*n*. A vessel whose diameter is included in the following limits is considered to belong to this order: [(D*n* − 1 + Δ*n* − 1) + (D*n* −Δ*n*)]/2 to the left and [(D*n* + Δ*n*) + (D*n* + 1 + Δ*n* + 1)]/2 to the right. Based on the values of the diameters and lengths measured, an order number is assigned to the vessel taken into consideration, for example order number 1. We then proceeded to measure the vessels of greater caliber and, with the same procedure, assigned a higher order, for example *n* + 1 and so on for all orders. At the end of the order assignments, the system performs a statistical comparison between the different ranges of diameters obtained, and if this comparison shows significance between the ranges of the values considered, the orders attributed to the vessels are correctly assigned; otherwise, the order assignment must be reviewed. The mathematical process was usually repeated at least twice to obtain data convergence. Strahler’s classification model foresees the existence of “segments” (each blood vessel placed between two bifurcations) connected in series that function as a single tube in the overall hemodynamics of microcirculation; each tube is defined as an “element.” The ratio between the total number of vessel segments and the total number of vessel elements for each given order represents the segment/element ratio (S/E). When this ratio assumes a value equal to 1, it indicates complete symmetry in the bifurcations; on the contrary, different ratios from 1 indicate bifurcation asymmetry.

Moreover, for a better assessment of ramification branchings, we elaborated a connectivity matrix where the vessels of order *n* may spring from the vessels of order *n* + 1, *n* + 2, ……. In general, the component of which in row *n* and column *m* was the ratio of the total number of elements of order *n* sprung from the elements in order *m*. To obtain the matrix, firstly, we grouped all vascular branches into elements and then recorded for each element of order *m* the number of elements of order *m*, *m* − 1, *m* − 2, ……, that arose directly from that element.

To obtain the mean value and standard deviation (SD) of each component of the matrix, we used statistics elaboration. The cross-sectional area was evaluated following the method described by Wiedman (1963).

### Analysis of Arteriolar Vasomotion

A computer-assisted power spectrum method based on a generalization short-time Fourier transform (GSTFT) was utilized to evaluate the rhythmic diameter changes of the arterioles (Varanini et al., 1998; Pradhan and Chakravarthy, 2011; Varanini, 2011). This analysis produces multi-resolution data and allows us to identify the multiple frequency components in the tracings of diameter variations, using a Hamming window based on frequency resolution. This procedure allows us to evaluate non-stationary data as the rhythmic changes in vessel diameters (Rossi et al., 2005). The analysis has been improved to detect the different frequency components: 0.005–0.0095 Hz [ultra-low frequency (ULF), nitric oxide (NO)-independent endothelial]; 0.0095–0.02 Hz [very low frequency (VLF), NO-dependent endothelial]; 0.02–0.06 Hz [intermediate low frequency (ILF), neurogenic]; 0.06–0.2 Hz [low frequency (LF), myogenic]; 0.2–2.0 Hz [high frequency (HF), respiratory activity]; and 2.5–4.5 Hz [very high frequency (VHF), cardiac activity] (Lapi et al., 2017).

### Evaluation of Endothelial and Inducible Nitric Oxide Synthase Expression by Western Blotting Analysis

Samples of cortex and striatum tissues from young and adult SHRs were subjected to homogenization (UltraTurrax, IKA, Staufen, Germany) in RIPA buffer containing antiprotease and antiphosphatase inhibitor cocktails; the protein concentration was determined by a Bio-Rad protein assay kit. Equal amounts of proteins were separated on 8% SDS-PAGE under reducing conditions and subsequently transferred to polyvinylidene difluoride (PVDF) membranes (Bio-Rad, Hercules, CA, United States). Following blocking, the membranes were incubated with specific antibodies at 4°C overnight, washed, re-incubated with a 1:3,000 dilution of horseradish peroxidase (HRP)-conjugated IgG secondary antibody (Santa Cruz Inc., Dallas, TX, United States), washed again, and the bands visualized by an electrochemiluminescence (ECL) chromogenic substrate (Bio-Rad). The Chemi Doc Imaging System (Bio-Rad) was utilized to detect the optical density of the bands and normalized to the optical density of a-tubulin using Image Lab software (Bio-Rad). To examine proteins of similar molecular weights, the PVDF membrane was subjected to a mild stripping protocol as recommended by Abcam (Cambridge, United Kingdom).

The specific antibodies used were: anti-eNOS (mouse monoclonal antibody, 1:500, ab76198; Abcam), anti iNOS (mouse monoclonal antibody, 1:1,000, ab49999; Abcam), and anti-α-tubulin (T-5168, 1:5,000; Sigma-Aldrich, St. Louis, MO, United States).

### Statistical Analysis

All data were reported as the mean ± SEM. Data were tested for normal distribution with the Kolmogorov–Smirnov test. One-way ANOVA for repeated measures was used to analyze the pial arteriolar diameter changes, while Dunnett’s multiple comparison test was used for *post hoc* analysis. The data elaborated by the spectral analysis were analyzed using the Wilcoxon and Man–Whitney tests.

Statistical analysis was carried out using the GraphPad Prism statistical package (version 4.0; GraphPad Software Inc., San Diego, CA, United States). Statistical significance was agreed at *p* < 0.05.

## Results

### Geometric Characteristics of the Pial Arteriolar Network

**FIGURE 1:**
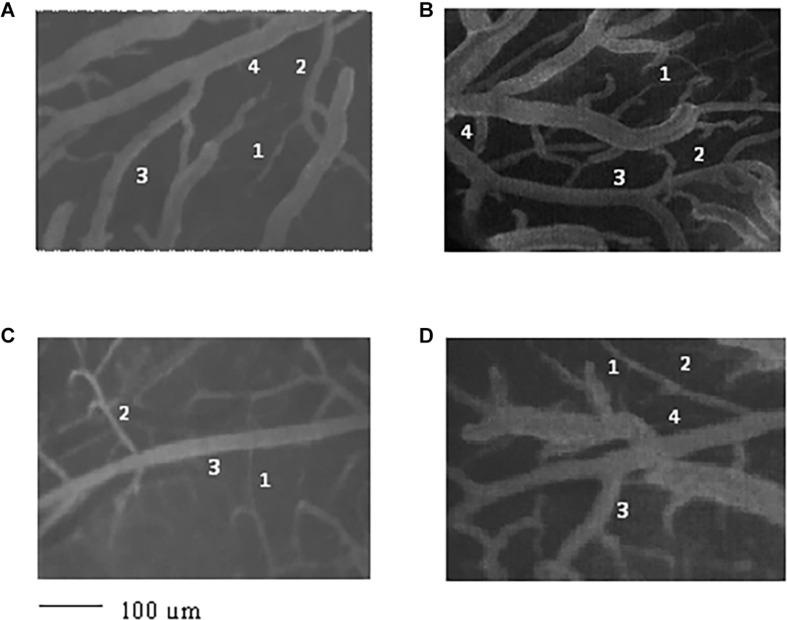
Image of the pial microvascular network of young spontaneously hypertensive rats (SHRs) **(A)**, young normotensive age-matched rats **(B)**, adult SHRs **(C)**, and adult normotensive age-matched rats **(D)**, obtained through a fluorescence microscope equipped with specific dedicated software. The different orders of arterioles are indicated with *1*, *2*, *3*, and *4*.

Strahler’s method, modified according to the diameter, was applied to classify the arterioles of the pial microcirculation of SHRs (Lapi et al., 2008). Young SHRs showed four orders of vessels according to diameter, length, and branchings. The classification started from capillaries to which order 0 was assigned; subsequently, to the arteries, which give rise to the capillaries, order 1 was given, and larger orders were gradually given up to order 4, which has the largest vessels we found in our preparations (Table 1 and Figure 1A).

**TABLE 1:** Classification of the pial arterioles in orders by Strahler’s method modified according to the diameters of young spontaneously hypertensive rats (SHR) and age-matched normotensive rats and of adult SHRs and age-matched normotensive rats.

Order	Arterioles (*n*)	Diameter (μm)	Length (μm)	Rat (*n*)
**Young SHRs**				

4	27	44.5 ± 3.0*	985 ± 124	15
3	102	32.0 ± 3.1*	512 ± 96	15
2	118	22.7 ± 2.8*	354 ± 85	15
1	135	17.3 ± 1.5*	128 ± 62	15

**Age-matched normotensive rats**				

4	30	43 ± 4*	848 ± 223	15
3	107	35.0 ± 2.8*	560 ± 180	15
2	120	26.5 ± 2.4*	355 ± 98	15
1	140	17 ± 2*	200 ± 45	15

**Adult SHRs**				

4				15
3	18	30.7 ± 2.4*	498 ± 87	15
2	45	21.4 ± 1.2*	305 ± 56	15
1	96	16.8 ± 0.7*	223 ± 38	15

**Age-matched normotensive rats**				

4	31	45.0 ± 3.8*	913 ± 200	15
3	106	32.0 ± 3.5*	478 ± 110	15
2	123	24.5 ± 3.0*	325 ± 100	15
1	138	15.0 ± 1.5*	188 ± 73	15

*p < 0.01 vs. different order.

**FIGURE 2:**
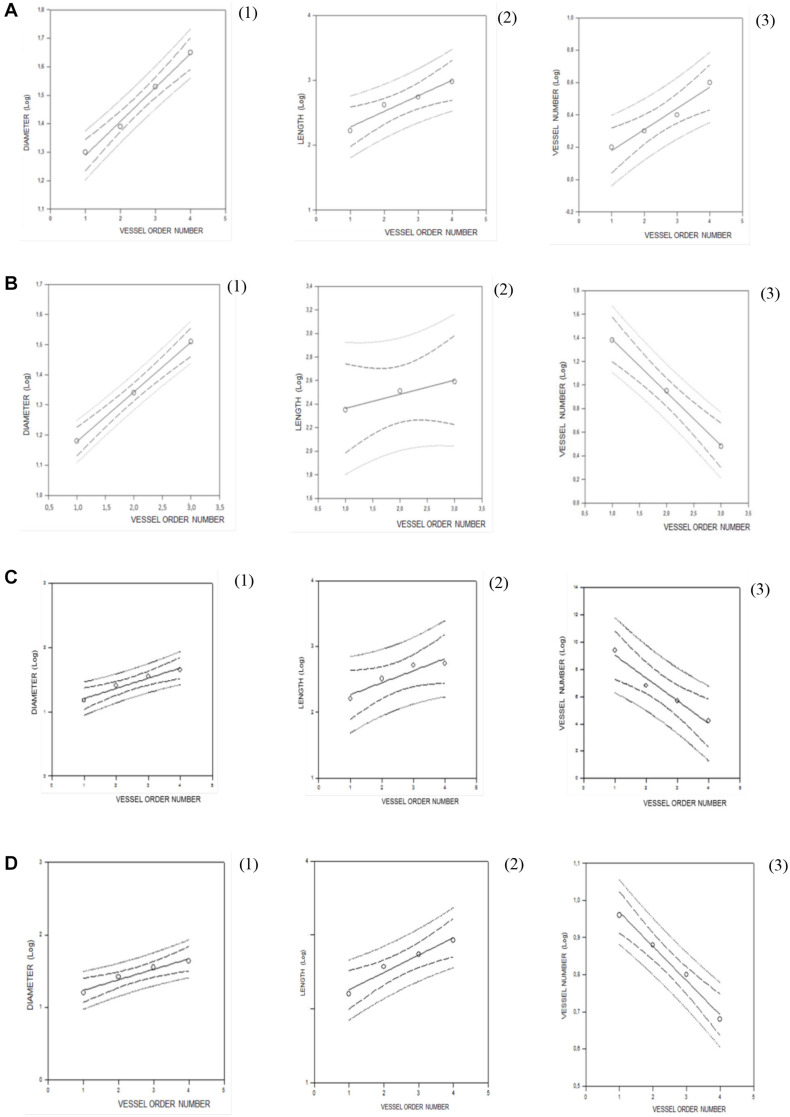
Regression lines developed from relationships between the mean diameter logarithm (*1*), length logarithm (*2*), logarithm of the vessel element number in successive orders of vessels (*3*), and arteriolar order number for young spontaneously hypertensive rats (SHRs) **(A)**, young normotensive age-matched rats **(B)**, adult SHRs **(C)**, and adult normotensive age-matched rats **(D)**.

Collecting data on the diameter, length, and branching number obtained from 15 animals, we observed that they obeyed Horton’s law (Table 2 and Figure 2A):

**TABLE 2:** Values of the empirical constants *a* and *b* that described the semilogarithmic relationships between the mean diameter, length, number of vessel elements, and order number of arterioles in young spontaneously hypertensive rats (SHRs) and age-matched normotensive rats and in adult SHRs and age-matched normotensive rats.

	Diameter Log_1__0_D*n* = *a* + *bn*	Length Log_1__0_L*n* = *a* + *bn*	Arteriolar number Log_1__0_N*n* = *a* + *bn*
**Young SHRs**			

*a*	1.170	2.040	0.050
*b*	0.119	0.240	0.130
*R*^2^	0.993	0.952	0.966
Ratio	1.32	1.74	1.35

**Age-matched normotensive rats**			

*a*	1.045	2.085	1.070
*b*	0.159	0.183	−0.170
*R*^2^	0.967	0.887	0.967
Ratio	1.44	1.52	0.68

**Adult SHRs**			

*a*	1.338	2.755	0.511
*b*	0.178	0.371	−0.140
*R*^2^	0.968	0.991	0.953
Ratio	1.51	2.35	1.38

**Age-matched normotensive rats**			

*a*	1.090	2.020	1.060
*b*	0.145	0.236	−0.092
*R*^2^	0.960	0.964	0.989
Ratio	1.40	1.72	0.81

*The data reported for a and b were derived from 15 animals each. The ratio is that between successive orders. R^2^ represents the correlation coefficient.*

Log_1__0_D*n* = *a* + *bn*;

Log_1__0_L*n* = *a* + *bn*; and

Log_1__0_N*n* = *a* + *bn*.

Another feature of pial microcirculation was the vessel arrangement in parallel or in series of arterioles, described by the S/E ratio which represents the total number of vessel segments divided by the total number of vessel elements in any given order. If the ratio was equal to 1, then the bifurcations were symmetrical, while values higher than 1 indicated asymmetric bifurcations. As reported in Table 3, order 4 arterioles showed asymmetric bifurcations, while the vessels belonging to lower orders had more symmetrical bifurcations.

**TABLE 3:** Segment and element ratios in each order of arterioles in young spontaneously hypertensive rats (SHRs) (A), in age-matched normotensive rats (B), in adult SHR (C) and in age-matched normotensive rats (D).

Order	S/E ratio	*N*
**Young SHRs**		

4	3.7	27
3	2.5	102
2	2.2	118
1	1.7	135

**Age-matched normotensive rats**		

4	3.55	30
3	2.12	107
2	1.63	120
1	1.20	140

**Adults SHRs**		

4		
3	1.2	18
2	1.9	45
1	1.7	96

**Age-matched normotensive rats**		

4	3.42	31
3	1.95	106
2	1.47	123
1	1.10	138

*S/E is the ratio between the total number of segments in a specified order and the total number of elements in that order. This ratio also represents the average number of vessel segments arranged in series for each order of arterioles. N is the number of vessels studied for each order.*

The connectivity matrix showed that order 4 arterioles gave origin to six order 4 vessels (0.22 × 27), 55 order 3 vessels (2.03 × 27), 41 order 2 vessels (1.53 × 27), and 12 order 1 vessels (0.43 × 27). No capillary originated from order 4 vessels. Order 3 arterioles gave rise mainly to vessels of order 2, order 2 vessels gave rise to many order 1 vessels and a few capillaries, while order 1 vessels gave origin principally to capillaries (Table 4).

**TABLE 4:** Connectivity matrix in young spontaneously hypertensive rats (SHRs) and in age-matched normotensive rats.

Young spontaneously hypertensive rats				
**Order**	***N***			

4	27			
3	102			
2	118			
1	135			

**Order *n***	**Order *m***			
	**1**	**2**	**3**	**4**

0	2.28 ± 0.22	0.25 ± 0.10	0	0
1	0.41 ± 0.18	2.37 ± 0.33	0.74 ± 0.22	0.43 ± 0.15
2	0	0.18 ± 0.10	2.05 ± 0.45	1.53 ± 0.66
3	0	0	0.58 ± 0.25	2.03 ± 0.75
4	0	0	0	0.22 ± 0.09

**Age-matched normotensive rats**				

**Order *n***	**Order *m***			
	**1**	**2**	**3**	**4**

0	2.41 ± 0.33	0.23 ± 0.09	0	0
1	0.37 ± 0.22	2.55 ± 0.24	0.82 ± 0.30	0.38 ± 0.17
2	0	0.22 ± 0.13	2.13 ± 0.51	1.62 ± 0.85
3	0	0	0.64 ± 0.37	2.18 ± 1.05
4	0	0	0	0.20 ± 0.11

**Order**	***N***			

4	30			
3	107			
2	120			
1	140			

*Young SHRs and age-matched normotensive rats showed four orders of pial arterioles. Values reported in the connectivity matrices represent the means ± SD. An element (n or m) that is in row n and column m is the ratio of the total number of elements of order m that sprung directly from the parent elements of order n divided by the total number of elements of order n. N is the number of vessels studied for each order of pial arterioles.*

Young normotensive age-matched rats showed an analog geometric arrangement compared to that observed in young SHRs. The pial arteriolar network was articulated into four vessel orders as reported in Table 1 and Figure 1B. The logarithm of the diameter or the length was directly proportional to the order of the vessels, and the ratios were 1.44, 1.52, respectively, while the logarithm of the branching number was inversely proportional to the vessel order number; the branching ratio was 0.68 (Figure 2B and Table 2). The smaller vessels were the most symmetrical (Table 3). Through the connectivity matrix, it was observed that order 4 originated six order 4 arterioles (0.20 × 30), 65 order 3 vessels (2.18 × 30), 49 order 2 vessels (1.62 × 30), 11 order 1 arterioles (0.38 × 30), and no capillaries. Order 3 arterioles gave origin to a greater number of order 2 vessels, those of order 2 to many vessels of order 1 and some capillaries, while the vessels of order 1 gave rise mainly to capillaries (Table 4).

Adult SHRs showed different geometric characteristics compared to young SHRs and adult normotensive age-matched rats. These rats displayed only three orders of vessels, as the largest arterioles in the microvascular field, reported in Table 1 and Figure 1C; however, the distribution in successive orders of arterioles followed Horton’s law. The logarithm of the diameter and length was directly proportional to the vessel order number, and the diameter and length ratios assumed values of 1.51 and 2.35, respectively (Table 2 and Figure 2C). The logarithm of the branching number was inversely proportional to the vessel order number, and the branching ratio was 1.38 (Table 2 and Figure 2C).

The ratio between the segments and elements indicated that the vessels originating more symmetrical branches were those of lower orders (1 and 2) in young SHRs, while those of order 3 were asymmetrical. In adult SHRs, order 3 arterioles were symmetrical, in contrast to the data detected in young SHRs (Table 3).

The data reported in the connectivity matrix indicated that from order 3 vessels originated five order 3 vessels (0.28 × 18), 24 order 2 vessels (1.35 × 18), and seven order 1 vessels (0.42 × 18).

Successively, order 2 gave origin mostly to order 1 vessels and a few capillaries, while order 1 vessels originated the capillaries (Table 5).

**TABLE 5:** Connectivity matrix of the pial arterioles in adult SHR (C) and in age-matched normotensive rats (D).

Adult spontaneously hypertensive rats				
**Order**	***N***			

3	18			
2	45			
1	96			

**Order *n***	**Order *m***			
	**1**	**2**	**3**	**4**

0	1.32 ± 0.45	0.12 ± 0.06	0	0
1	0.27 ± 0.08	1.58 ± 0.33	0.42 ± 0.21	0
2	0	0.12 ± 0.06	1.35 ± 0.75	0
3	0	0	0.28 ± 0.11	0
4	0	0	0	0

**Age-matched normotensive rats**				

**Order**	***N***			

4	31			
3	106			
2	123			
1	138			

**Order *n***	**Order *m***			
	**1**	**2**	**3**	**4**

0	2.50 ± 0.27	0.20 ± 0.12	0	0
1	0.42 ± 0.31	2.43 ± 0.27	0.80 ± 0.25	0.45 ± 0.12
2	0	0.24 ± 0.13	2.08 ± 0.35	1.58 ± 0.75
3	0	0	0.73 ± 0.41	2.10 ± 0.90
4	0	0	0	0.17 ± 0.15

*Adult SHRs showed only three orders of pial arterioles, while age-matched normotensive rats showed four. Values reported in the connectivity matrices represent the means ± SD. An element (n or m) that is in row n and column m is the ratio of the total number of elements of order m that sprung directly from the parent elements of order n divided by the total number of elements of order n. N is the number of vessels studied for each order of pial arterioles.*

Adult normotensive age-matched rats showed four orders of vessels, as observed in young normotensive age-matched rats (Table 1 and Figure 1D). The distribution in successive orders of vessels obeyed Horton’s law. The logarithm of the diameter and length was directly proportional to the vessel order number (diameter and length ratios: 1.40 and 1.72, respectively) (Table 2 and Figure 2D), while the logarithm of the branching number was inversely proportional to the vessel order number (branching ratio = 0.81) (Table 2 and Figure 2D). The greatest branching asymmetry was observed in the major orders 4 and 3 (Table 3).

Moreover, the connectivity matrix showed the same trend observed in the connectivity matrix elaborated from the data of young normotensive age-matched rats (Table 5).

It is worth noting that the decrease in the number of branchings and vessel orders in the microvascular field, observed in adult SHRs compared to young SHRs, involved a significant reduction in the overall cross-sectional area (CSA) by 75.0 ± 6.5%, indicating a significant increase in the peripheral resistance of the terminal arteriolar networks. When we compared the single orders between young and adult SHRs, we observed that order 3 arteriolar CSA decreased by 83.8 ± 6.8% in adult vs. young rats; in order 2 vessels, there was a reduction of 65.8 ± 3.5%, while the reduction in order 1 arteriolar CSAs was of 32.9 ± 2.5%. Moreover, when we evaluated the overall trend in the three orders of arterioles, we obtained an overall reduction in CSA of 68.4 ± 4.5% in adult SHRs.

In normotensive age-matched rats, we observed a slight reduction in the overall cross-sectional area when comparing young with adult rats of 11.3 ± 1.7%. The data were even more relevant when we compared adult SHRs with adult normotensive age-matched rats: the overall CSA decreased by 69.6 ± 5.2%.

### Vasomotion Evaluation

Young and adult SHRs showed six frequency components detected in 30-min recordings.

**FIGURE 3:**
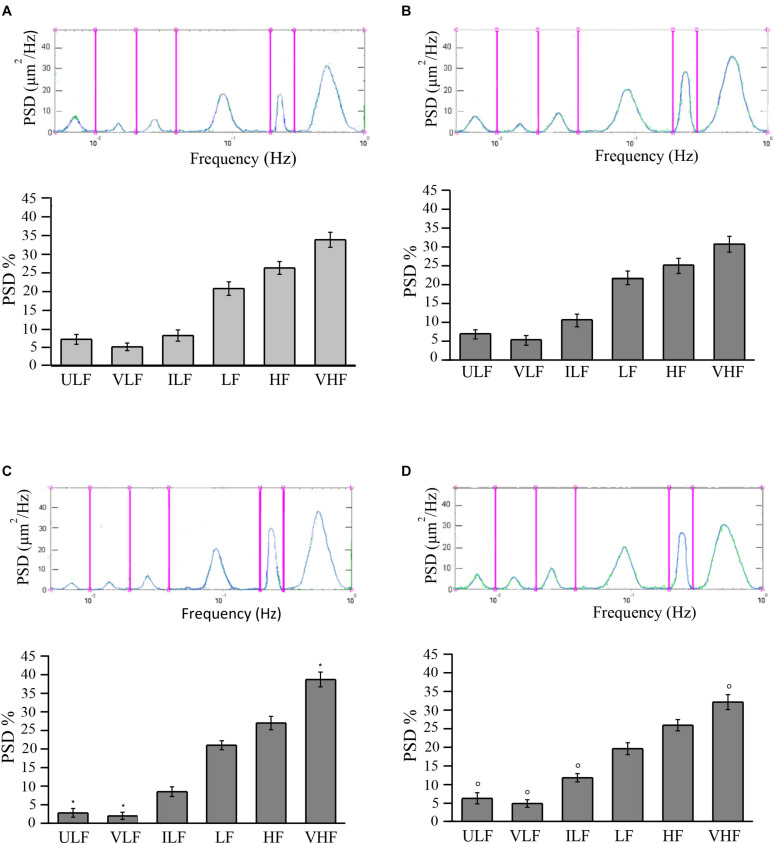
Rhythmic diameter changes (*upper*) of order 3 arterioles and the six corresponding frequency components (*bottom*) expressed as percent normalized power spectral density (PSD, in square micrometers per hertz) in young spontaneously hypertensive rats (SHRs) **(A)** and in young normotensive age-matched rats **(B)**. Adult SHRs showed a significant decrease of the frequency components related to endothelial activity (ULF and VLF) compared with those detected in young SHRs. In **(C,D)** are the rhythmic diameter changes (*upper*) of order 3 arterioles and the six corresponding frequency components (*bottom*) expressed as percent normalized PSD (in square micrometers per hertz) in adult SHRs and in adult normotensive age-matched rats, respectively. Young and adult normotensive age-matched rats showed no significant change of all six frequency components when compared with each other, while there was a significant difference in ULF, VLF, ILF, and VHF when compared with adult SHRs. *ULF*, ultra-low frequency; *VLF*, very low frequency; *ILF*, intermediate low frequency; *LF*, low frequency; *HF*, high frequency; *VHF*, very high frequency. *p < 0.01, significantly different from the corresponding values observed in young SHRs; °*p* < 0.01, significantly different from the corresponding values observed in adult SHRs.

In young SHRs, the distribution of the different frequencies, quantified by the power spectral density, revealed that the first two frequencies correlated to endothelial activity (ULF and VLF) represented 7.0 ± 1.3 and 5.0 ± 1.0%, respectively, of the total power density. Of the total spectral density, 8.0 ± 1.5% was due to the third component (ILF), 20.5 ± 1.8% to the fourth component (LF), while 26.0 ± 1.7 and 33.5 ± 2.0% corresponded to the fifth (HF) and sixth (VHF) components of the spectrum, respectively (Figure 3A).

In adult SHRs, spectral analysis indicates that the first two frequency components related to endothelial activity were significantly reduced (ULF and VLF), both below 3% of the total spectral power, on the average. On the other hand, the component related to heart rate (VHF) increased by more than 5% of the total power density, on average, when compared to the values detected in young SHRs (Figure 3B).

Young normotensive age-matched rats showed no significant changes in the different frequencies when compared to young SHRs (Figure 3C) or even to adult normotensive age-matched rats (Figure 3D). The first two frequency components (ULF and VLF) represented 6.8 ± 1.2 and 6.2 ± 1.5% in young normotensive age-matched rats and 5.2 ± 1.3 and 4.8 ± 1.0% in adult normotensive age-matched rats, respectively, of the total spectral density. ILF was 10.5 ± 1.7 and 11.7 ± 1.1% of the total spectral density in the two groups, respectively, LF was 21.8 ± 1.8 and 19.5 ± 1.6% of the total spectral density in young and adult normotensive age-matched rats, respectively. The last two frequency components (HF and VHF) represented 25.0 ± 2.0 and 30.7 ± 2.1%, respectively, in young normotensive age-matched rats and 25.8 ± 1.5 and 32.0 ± 2.0% in adult normotensive age-matched rats, respectively, of the total spectral density.

### eNOS and iNOS Expression

**FIGURE 4:**
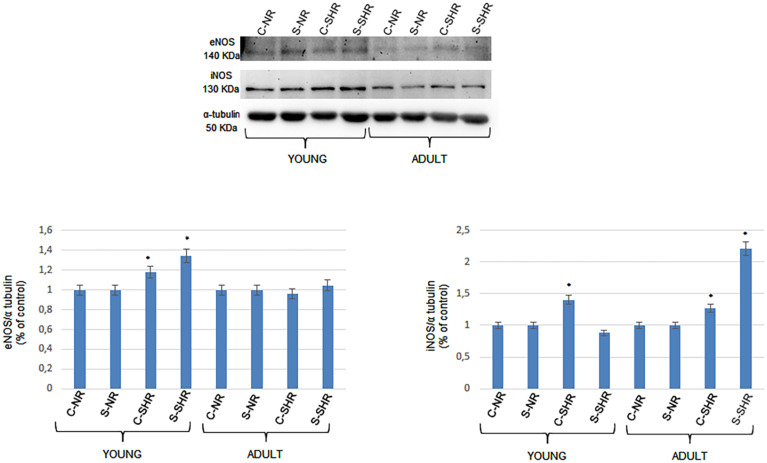
Western blotting of endothelial (eNOS) and inducible nitric oxide synthase (iNOS) expression in two cerebral zones (cortex and striatum) in young age-matched normotensive rats, in young spontaneously hypertensive rats (SHRs), in adult age-matched normotensive rats, and in adult SHRs. *Bottom*: corresponding densitometric values (mean ± SD). *C-NR*, cortex of normotensive rats; *S-NR*, striatum of normotensive rats; *C-SHR*, cortex of SHRs; *S-SHR*, striatum of SHRs. *p < 0.01 vs. age-matched normotensive rats.

We analyzed the expressions of endothelial (eNOS) and inducible nitric oxide synthase (iNOS) in young and adult SHRs and in normotensive age-matched rats by Western blotting. The eNOS levels significantly changed in both the cortex and striatum in young SHRs compared to normotensive age-matched rats. On the other hand, in adult SHRs, there were no significant differences in the cortex and striatum when compared to the values in adult normotensive age-matched rats. Furthermore, iNOS expression was significantly different when comparing the different groups. In young SHRs, indeed, the iNOS levels in the cortex showed significant increases compared to normotensive age-matched rats. In adult SHRs, iNOS significantly increased in the cortex and the striatum when compared to normotensive age-matched rats; the increase was higher in the striatum (Figure 4).

## Discussion

The data obtained in the present study indicate the different geometric arrangements of pial microcirculation when systemic arterial pressure had increased with aging.

Comparing young SHRs, without hypertension, to adult SHRs, with an overt hypertensive state, an overall remodeling of the pial arteriolar networks has been observed. Young SHRs presented four orders of vessels, the same pattern as observed in normotensive rats (Lapi et al., 2008); conversely, in adult SHRs, only three orders of vessels were detected. In particular, adult SHRs showed a reduction in the total number of arterioles in the terminal networks: on average, the number of vessels in adult SHRs decreased by 58.3% compared to those detected in young SHRs. Consequently, there was a significant reduction in the cross-sectional area of the terminal arteriolar system by 75.1 ± 5.2%, indicating a significant increase in peripheral arteriolar resistance. Therefore, it is reasonable to suggest that the development of high blood pressure levels was accompanied by a dramatic decrease in the arteriolar cross-sectional area due to the remodeling of networks characterized by a reduction of the largest vessels (order 4 not revealed) in the microscopic field observed. Substantially, microvascular rarefaction affects all orders of the vessels.

Another result obtained by the geometric classification of arteriolar vessels was the degree of symmetry of the branchings obtained by calculating the ratios between segments and elements. In young SHRs, the most symmetrical vessels were those of order 1, as noted in normotensive rats (Lapi et al., 2008), while in adult SHRs were those of order 3. This means that order 3 vessels presented a smaller number of branching vessels in adult SHRs when compared with those evaluated in young SHRs.

Finally, from the elaboration of the connectivity matrix, it is worth noting that, in all groups of animals studied, the higher-order vessels gave origin mostly to vessels of lower order. This trend is in accordance with that shown in normotensive rats previously described (Lapi et al., 2008).

Our study reports significant changes in the geometric characteristics of the arteriolar networks, indicating that redistribution of blood flow at the levels of arteriolar terminal branchings was different in young and adult SHRs, with consequent different blood supplies to the brain tissue. Up to now, the remodeling of the arteriolar networks during hypertension (Pries et al., 2005; Feihl et al., 2008) has been described, but without paying attention to the general new arrangements of the microvascular structures involved in the blood flow supply to the tissues. Therefore, hypertension was accompanied by a remodeling of the arteriolar vessels, resulting in the reduction of the lumen diameter, an increase in the wall-to-lumen ratio, as reported by several authors, and a significant change in the geometric characteristics of the pial arteriolar networks.

Previous studies have indicated that vascular and cerebrovascular changes during hypertension are often linked to inflammatory processes. Our data indicate that, in the young and adult SHRs, iNOS expression significantly increases in the cortex in young and in both the cortex and the striatum in adult SHRs. These data, therefore, demonstrate that the increase in arterial blood pressure and the consequent changes in the geometric features of the cerebral arteriolar networks are accompanied by stimulation of iNOS expression, contributing to trigger the inflammatory cascades, as reported in previous studies (Laroux et al., 2001; Soufli et al., 2016). The main molecules involved in inflammatory processes are pro-inflammatory interleukins (IL-1b and IL-6) and tumor necrosis factor alpha (TNF-α), which were significantly increased in the brain tissue of adult SHRs (Tayebati et al., 2016; Avolio et al., 2018).

The inflammatory mechanisms are known to affect vascular walls, leading to blood–brain barrier (BBB) dysfunction; consequently, the inflammation could result in damage of the endothelial cells and extravascular edema with consequent injury to the adjacent brain tissues.

The structural changes reported in the present study were accompanied by variations in arteriolar vasomotion. The study of the components influencing the rhythmic diameter changes of the arterioles indicates that, in adult SHRs, the components related to endothelial activity were significantly reduced compared to those observed in young SHRs. These alterations demonstrate that changes in arteriolar walls could involve endothelial cells, which are unable to produce or release the dilating factors.

However, the expression of eNOS did not change in adult SHRs when compared to normotensive age-matched rats, while eNOS expression was higher in young SHRs compared to normotensive age-matched rats. These data are accompanied by the finding of a reduced power spectral density of the endothelial-related frequency components in adult SHRs, while in young SHRs there were no significant changes in the power spectral density of the components related to endothelial cell-derived factors when compared to both young and adult normotensive age-matched rats. However, in adult SHRs, we found that iNOS expression was higher in both the cortex and the striatum.

Our results indicate that arteriolar vasomotion is related to several frequency components differently affected during the development of blood arterial hypertension. In particular, eNOS expression in the cortex and striatum of young SHRs significantly increased compared to normotensive age-matched rats, while iNOS expression increased only in the cortex. However, we did not detect a decrease in the power spectral density of the frequency components related to endothelial cell-derived factors in young SHRs. On the other hand, in adult SHRs, we found an increase in iNOS expression in the striatum and a decrease in the power spectral density of the frequency components related to endothelial cell-derived factors. These data may suggest that inflammation triggered by iNOS could affect endothelial cells, reducing the release or the formation of endothelial cell-related factors. However, further studies are required to clarify the complex mechanisms of network remodeling and the related changes in the arteriolar functions in SHRs.

## Conclusion

In conclusion, hypertension causes a progressive change in cerebral blood flow supply, dependent not only on a different structure of the arteriolar walls but also on a different microarchitecture of the arteriolar networks with a smaller number of vessels and reduced conductivity, characteristic of rarefaction. This was accompanied in our model by the reduction in the formation or release of endothelial cell-related factors regulating arterial vasomotion.

## Data Availability Statement

The raw data supporting the conclusions of this article will be made available by the authors, without undue reservation.

## Ethics Statement

The animal study was reviewed and approved by the University of Pisa and Italian Health Ministry (Permit Number: 156/2017- PR).

## Author Contributions

DL, MD, MC, AC, and BG designed and supervised the study. DL, MD, MC, and AC performed the experiments. LS performed the experiments and analyzed the data. DL, NS, MV, RS, and AC analyzed the data. All authors contributed to the article and approved the submitted version.

## Conflict of Interest

The authors declare that the research was conducted in the absence of any commercial or financial relationships that could be construed as a potential conflict of interest.
